# Meteorological drivers and short-term prediction of dengue fever in a tropical region of China: evidence from Hainan Province

**DOI:** 10.3389/fpubh.2026.1919479

**Published:** 2026-08-28

**Authors:** Yiyao Lian, Yan Jin, Yufei Wang, Li Qiu, Jingjing Chen, Yuqing Guo, Rui Jiang, Dapeng Yin, Wenbiao Hu, Liping Wang

**Affiliations:** 1 National Key Laboratory of Intelligent Tracking and Forecasting for Infectious Diseases, Division of Infectious Disease, Chinese Center for Disease Control and Prevention & Chinese Academy of Preventive Medicine, Beijing, China; 2 National Institute for Nutrition and Health, Chinese Center for Disease Control and Prevention & Chinese Academy of Preventive Medicine, Beijing, China; 3 Hainan Provincial Center for Disease Control and Prevention (Hainan Academy of Preventive Medicine), Haikou, China; 4 Office of General Administration, Chinese Center for Disease Control and Prevention & Chinese Academy of Preventive Medicine, Beijing, China; 5 Department of Automation, Tsinghua University, Beijing, China; 6 Ecosystem Change and Population Health Research Group, School of Public Health and Social Work, Queensland University of Technology, Brisbane, QLD, Australia

**Keywords:** dengue fever, distributed lag non-linear models, early risk prediction index, meteorological factors, short-term prediction

## Abstract

**Objective:**

Meteorological factors influence dengue transmission through nonlinear and lagged effects. This study assessed these associations in Hainan Province and developed an early risk prediction index.

**Methods:**

Weekly dengue case and meteorological data from Hainan Province (2018–2019 and 2023–2024) were analyzed using distributed lag non-linear models (DLNMs). An early risk prediction index(ERPI) was constructed from DLNM-derived cumulative meteorological risk signals and imported cases. Predictive performance was evaluated using leave-one-year-out cross-validation and assessed by the area under the receiver operating characteristic curve (AUC), sensitivity, specificity, positive predictive value (PPV), and negative predictive value (NPV).

**Results:**

Among 927 dengue cases reported during the four modelled years, 727 (78.43%) were locally acquired. Weekly mean daily maximum temperature (Tmax), precipitation (Pmean), wind speed (WSmean), and diurnal temperature range (DTRmean) showed nonlinear, lag-dependent associations with dengue risk, with distinct temporal patterns. Higher Tmax showed longer-lag effects, peaking at 33.73 °C at lag 8 weeks (*RR* = 2.45, 95% CI: 1.70–3.63). Its cumulative effect was strongest over lags 5–8 weeks, peaking at approximately 32 °C. Pmean showed a nonlinear association, peaking at 4 mm/day at a lag of 4 weeks (*RR* = 1.92, 95% CI: 1.59–2.31), with significant cumulative effect across both the 1–4- and 5–8-week windows. Low WSmean and moderate DTRmean showed their strongest cumulative associations over lags 1–4 weeks, peaking at 2.0 m/s and 6.1 °C, respectively. These harmful associations attenuated over lags 5–8 weeks, whereas higher WSmean and DTRmean were generally protective. The ERPI predicted local dengue occurrence within the subsequent 1–4-week windows, with AUCs of 0.762–0.843. Across these windows, sensitivities were 0.768–0.797, specificities were 0.644–0.708, PPVs were 0.468–0.624, and NPVs were 0.822–0.911.

**Conclusion:**

In Hainan, high temperature was primarily associated with medium-to-long-term dengue risk, whereas low-to-moderate diurnal temperature range increased short-term risk. Precipitation was associated with risk across both short and medium-to-long lags, while cumulative wind-speed associations were strongest over shorter lags and attenuated thereafter. The DLNM-based early risk prediction index may support meteorology-informed early warning and dengue control in tropical settings, although requiring external validation is needed before operational implementation.

## 1 Introduction

Dengue is an acute infectious disease caused by the dengue virus and transmitted to humans primarily through the bites of *Aedes aegypti* and *Aedes albopictus*. Across tropical and subtropical regions, it now spreads faster than any other mosquito-borne viral disease (1). In 2024, the World Health Organization reported more than 14.6 million dengue cases and over 12,000 associated deaths worldwide (2). Over the past few decades, climate change, rapid urban growth, and rising international trade and travel have pushed dengue into new regions and sped up its spread, and it now represents a serious public health concern worldwide. Accordingly, dengue has been listed by WHO as a neglected tropical disease and a priority for the 2021–2030 global public health agenda (1, 3). Dengue transmission arises from the interplay of entomological, immunological, and socioeconomic factors, yet meteorological conditions remain among its most fundamental drivers.

Temperature, rainfall, and relative humidity affect the survival, reproduction, and biting activity of the vector, and in doing so shape how efficiently the virus is transmitted (4–6). Specifically, temperature affects key mosquito life-history traits and vector competence and may also influence dengue virus structure (7); precipitation determines the availability of aquatic breeding habitats (8); and high relative humidity prolongs mosquito survival and raise population density, facilitating spread (9). These effects are frequently nonlinear and delayed, with reported lags of a few weeks to several months (10, 11). Because the shape, thresholds, direction, and timing of these relationships differ considerably between regions, evidence from one location cannot be applied directly to another (12–15). Jointly characterizing the nonlinear and lagged effects of meteorological factors within a specific region is therefore essential for identifying critical exposure windows and for developing locally tailored early-warning systems.

In China, dengue epidemiology is driven by imported infections that seed local outbreaks (16), with transmission concentrated in Yunnan, Guangdong, and Hainan (17), and 86.6% of imported cases originating from Southeast Asian countries such as Cambodia and Myanmar (18). Among these regions, Hainan is particularly vulnerable: as a tropical island at the southernmost tip of China, its consistently high temperatures, high relative humidity, abundant rainfall, and dense vegetation sustain near year-round breeding of *Aedes* vectors, rendering the province exceptionally suitable for dengue transmission (19). In line with this, Hainan has seen local outbreaks grow more frequent and larger in scale over recent years (20). This risk is poised to intensify further: the full implementation of island-wide customs clearance in December 2025 will heighten population mobility and cross-border exchange, thereby raising the risk of imported infections and subsequent local transmission, while climate-change projections indicate growing dengue vulnerability in low-latitude coastal regions such as Hainan (21).

Previous studies have documented meteorological associations with dengue incidence across diverse regions (15, 22–24). However, few studies have systematically explored the nonlinear and lagged meteorological effects on locally acquired dengue transmission in Hainan’s unique tropical island setting, and even fewer have evaluated the utility of integrating meteorological variables with imported case data to assess the risk of local transmission. Moreover, most existing dengue prediction models adopt conventional regression methods with prespecified lag periods (11, 25), and therefore cannot fully capture heterogeneous meteorological impacts across varying exposure levels and lag windows or characterize continuous nonlinear cumulative effects. Importantly, the launch of island-wide special customs operations in the Hainan Free Trade Port in December 2025 may facilitate cross-border movement and consequently increase the risk of imported mosquito-borne diseases.

Accordingly, this study focuses on Hainan Province to systematically evaluate the nonlinear lagged effects of major meteorological factors on dengue incidence risk, with the aim of identifying critical exposure time windows and risk thresholds. Building upon these findings, we further integrated meteorological lag effects with information on imported cases to construct a dengue risk prediction index. This meteorology-based dynamic risk prediction framework tailored to Hainan provides a practical tool for dengue early warning and targeted import prevention and control for the Hainan Free Trade Port.

## 2 Materials and methods

### 2.1 Study site

Hainan Island, a tropical island in China (18°21′–20°21′N, 108°67′–111°27′E), is surrounded by the sea, facing Vietnam across the Beibu Gulf to the west and the Leizhou Peninsula (Guangdong) across the Qiongzhou Strait to the north. It has a tropical monsoon maritime climate with mild seasonal variation, an annual mean temperature of ~23 °C, annual precipitation >900 mm, and high relative humidity. These conditions favor dengue vector breeding, making Hainan a high-risk area for dengue in China (19, 26).

### 2.2 Data collection

#### 2.2.1 Dengue surveillance data

Dengue case data were obtained from the National Notifiable Disease Reporting System (NNDRS). We included all confirmed and clinically diagnosed dengue cases with symptom onset between 1 January 2018 and 31 December 2024. Because COVID-19-related interventions severely disrupted population mobility, case importation, health-care-seeking behavior, and local dengue transmission during 2020–2022, the exposure–response modelling was restricted to 2018–2019 and 2023–2024. Cases were classified as imported or locally acquired according to epidemiological investigation, primarily based on travel history during the 14 days before symptom onset and the likelihood of local transmission. Imported cases were defined as patients with a history of travel to dengue-endemic areas within 14 days before symptom onset, whereas locally acquired cases were defined as patients without such travel history during the same period (27). Locally acquired cases were used as the outcome in the exposure-response analyses. All records in the NNDRS undergo manual review by designated personnel without missing values.

#### 2.2.2 Meteorological data

Daily meteorological data were retrieved from the website Weather in 241 Countries Worldwide^1^ for stations in five cities in Hainan, including daily mean, maximum, and minimum temperature (°C), mean sea level pressure (mmHg), mean relative humidity (%), visibility (km), daily total precipitation (mm/day), mean wind speed (m/s), and extreme wind speed (m/s). The diurnal temperature range (DTR, °C) was calculated for each day as the difference between the daily maximum and minimum temperatures to capture within-day temperature variability. In this study, missing meteorological data at each site were imputed using multiple imputation by chained equations (MICE) (28). Ten imputed datasets were generated in total, and the results from all datasets were subsequently averaged to acquire the final imputed value for each missing observation (29). Station-level data were averaged to represent province-wide conditions, then aggregated to weekly metrics (weekly means and maxima).

### 2.3 Statistical analysis

#### 2.3.1 Descriptive analysis

We described the temporal distribution of locally acquired dengue and meteorological conditions in Hainan from 2018 to 2024. Weekly dengue counts and weekly summaries of each meteorological variable were aligned to a common epidemiological-week scale, and their co-variation was displayed using time-series plots; continuous meteorological variables were summarized as means (standard deviations) and percentiles. Because the stringent non-pharmaceutical interventions implemented for COVID-19 during 2020–2022 markedly disrupted local transmission and rendered the meteorology–dengue relationship atypical for those years, the exposure–response modelling was restricted to the 2018–2019 and 2023–2024 transmission seasons.

#### 2.3.2 Distributed lag non-linear models (DLNMs)

DLNMs were used to estimate the non-linear and delayed associations between meteorological factors and the weekly incidence of locally acquired dengue in Hainan. Through a bidimensional cross-basis function, this framework characterizes the exposure–response and lag–response relationships simultaneously and has been widely adopted in studies of meteorological effects on infectious diseases (30). Given the marked overdispersion in the weekly case series, the models were fitted within a generalized additive modelling framework with a quasi-Poisson family to account for the extra-Poisson variation (13).

Prior to multivariable modelling, collinearity among the meteorological factors was screened using pairwise Spearman correlation coefficients and variance inflation factors (VIFs), with thresholds of *|r|* > 0.70 and VIF > 5 used to flag strong correlation and substantial collinearity, respectively (Supplementary Figures S1, S2). When two factors were strongly correlated, the mean daily maximum temperature was retained in preference given its established mechanistic role in dengue transmission (22). The final models therefore included four factors: weekly mean daily maximum temperature (Tmax, °C), weekly mean daily precipitation (Pmean, mm/day), weekly mean daily wind speed (WSmean, m/s), and weekly mean diurnal temperature range (DTRmean, °C).

For each meteorological factor, the cross-basis was constructed using natural cubic splines in both the exposure–response and the lag dimensions, with the degrees of freedom (*df*) informed by previous studies and finalized by minimizing the quasi-Akaike information criterion (QAIC) (13, 31). Accordingly, 2 and 3 *df* were assigned to the exposure–response and lag dimensions, respectively, for Tmax, whereas 3 *df* were assigned to both dimensions for precipitation, wind speed, and diurnal temperature range. A common maximum lag of 8 weeks was applied to all factors, a window chosen to encompass the complete developmental (egg-to-adult) cycle of the *Aedes* vector together with the intrinsic and extrinsic incubation periods of dengue virus (32). In addition, sensitivity analyses were conducted by varying the maximum lag length specified in the model to verify the robustness of the research findings.

Separate models were fitted, with each factor in turn designated as the primary exposure: its cross-basis entered the model as the primary term, while the remaining three factors were adjusted for using natural cubic splines of their current-week values. Each model also adjusted for long-term trend, seasonality, and case importation. The DLNM was specified as shown in Equation 1:

$$\begin{array}{l} \log[E(Y_{t})]=\beta_{0}+\mathrm{cb}(X_{k,t})+\sum_{j}\mathrm{ns}(Z_{j,t},\;df_{j})\\ +\mathrm{ns}(\text{time},4)+s(\mathrm{wo}y_{t})+\text{Impor}t_{t-2} \end{array}$$ (1)

Here, $Y_{t}$ is the number of locally acquired dengue cases reported in Hainan in week *t*; $\beta_{0}$ is the intercept; $X_{k}$ is the meteorological factor of primary interest and *cb*(*X_k, t_*) is the cross-basis function over lags 0–8 weeks; $\mathrm{ns}(Z_{j,t},df_{j})\;$ are natural cubic splines of the current-week values of the remaining meteorological factors; ns(time,4) is a natural cubic spline of calendar time with 4 *df* controlling for long-term trend; $s(\mathrm{wo}y_{t})\;$ is a cyclic cubic spline of week of the year, with the *df* selected automatically, controlling for seasonality; and $\text{Impor}t_{t-2}$ is the cumulative number of imported cases over the two preceding weeks.

The median of each meteorological variable was used as the reference for calculating relative risks (*RRs*) and 95% confidence intervals (*CIs*). To quantify the influence of extreme conditions, the 5th and 95th percentiles of each factor were contrasted with the reference.

#### 2.3.3 Early dengue risk prediction index

For each meteorological factor, we extracted the contribution of its cross-basis to the linear predictor at week *t*, which aggregated the lag-specific effects of its exposure over the preceding 0 to 8 weeks into a single value, and used this quantity as the factor’s cumulative risk signal ( $Z_{i,t}^{\left(k\right)}$ , *i* = 1–4). To make the four signals comparable, each was standardized using the *Z*-score transformation, and the number of imported cases in the preceding 2 weeks was included after a log(x + 1) transformation. All predictors were derived using only information available up to week *t*.

For each prediction horizon *k* (*k* = 1, 2, 3, and 4 weeks), a separate logistic regression model was fitted using the same set of week-t predictors but a horizon-specific binary outcome, defined as the occurrence of at least one locally acquired dengue case during weeks *t + 1* to *t + k*:

$$\begin{array}{l} \text{logit}[P(Y_{t}^{\left(k\right)}=1)]=\beta_{0}^{\left(k\right)}+\sum_{i=1}^{4}\beta_{i}^{\left(k\right)}Z_{i,t}^{\left(k\right)}\\ +\gamma^{\left(k\right)}\log(1+\text{Impor}t_{t-2}) \end{array}$$ (2)

Here, $Y_{t}^{\left(k\right)}=1$ indicates the occurrence of any locally acquired dengue case in at least 1 week from (*t + 1*) to (*t + k*). $Z_{i,t}^{\left(k\right)}$ denotes the Z-standardized cumulative risk signal of meteorological factor *i* at week *t*; $\text{Impor}t_{t-2}$ is the number of imported cases in the two preceding weeks; and $\beta_{0}^{\left(k\right)}$ , $\beta_{i}^{\left(k\right)}$ , and $\gamma^{\left(k\right)}$ are horizon-specific coefficients.

The dengue risk prediction index for horizon k was defined as the predicted probability from Equation 2 and expressed as a percentage, as shown in Equation 3:

$${\text{ERPI}}_{t}^{\left(k\right)}=100\times P(Y_{t}^{\left(k\right)}=1)$$ (3)

The index ranges from 0 to 100 and can be interpreted directly as the percentage probability of at least one locally acquired dengue case occurring within the next *k* weeks.

This study adopted leave-one-year-out cross-validation to evaluate the predictive performance of the model. The cross-validation was divided into four folds; in each fold, 1 year was exclusively retained as the validation set, and the remaining 3 years served as the training set for model fitting. For each prediction horizon, out-of-sample predictions from all four folds were pooled to plot receiver operating characteristic (ROC) curves, and the optimal classification probability threshold was selected by maximizing the Youden index. At the optimal threshold, sensitivity, specificity, positive predictive value (PPV), and negative predictive value (NPV) were calculated to further assess the classification performance of the model. To verify the robustness of the results, a sensitivity analysis was conducted, in which the outcome indicator was redefined as the occurrence of a week with three or more locally confirmed cases within the subsequent *k* weeks, and the entire modeling and analytical workflow was replicated in full.

### 2.4 Analysis software

R software (version 4.5.1; R Foundation for Statistical Computing, Vienna, Austria) was used for data analysis and graphical visualization. The “dlnm” and “mgcv” packages were used to construct and fit the DLNMs (33, 34), with the cyclic cubic spline for seasonality implemented through “mgcv.” Natural cubic spline functions were specified using the “splines” package, and receiver operating characteristic (ROC) curve analysis was performed using the “pROC” package. All statistical tests were two-sided, and statistical significance was defined as *p* < 0.05.

## 3 Results

### 3.1 Epidemiological characteristics of dengue

**Figure 1:**
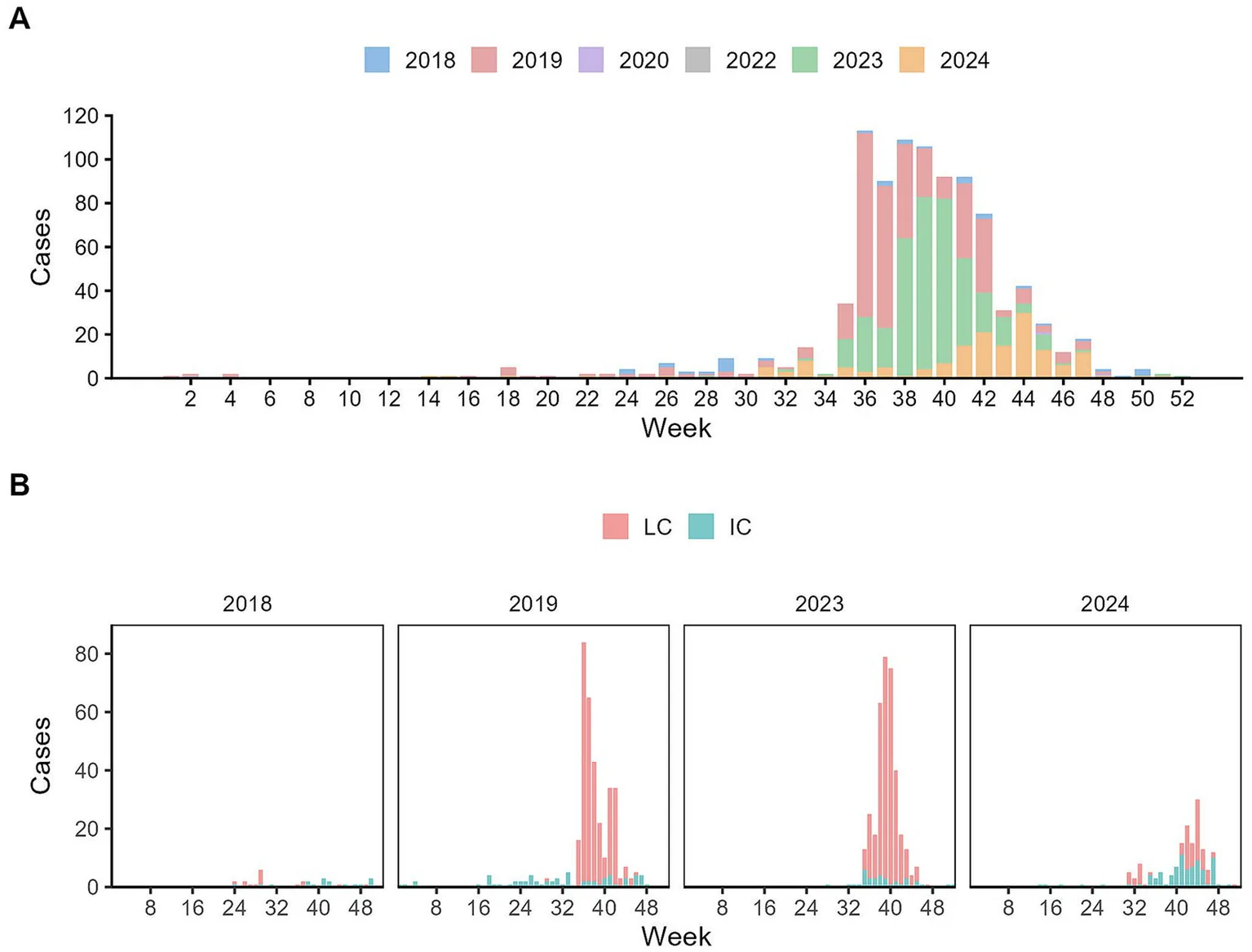
Temporal trends in locally acquired and imported dengue cases in Hainan Province, 2018–2024. **(A)** Overall trends of dengue cases from 2018 to 2024; **(B)** Locally acquired and imported dengue cases during 2018, 2019, 2023 and 2024. LC, locally acquired dengue case; IC, imported dengue case.

Between 1 January 2018 and 31 December 2024, 930 dengue cases were reported in Hainan Province. A total of 927 cases occurred in 2018, 2019, 2023, and 2024, including 727 locally acquired cases (78.43%) and 200 imported cases (21.57%). The temporal distribution was clearly phased, with two major peaks in 2019 and 2023 (370 and 363 cases, respectively), whereas almost no cases were reported during 2020–2022. Locally acquired dengue showed a typical summer-autumn seasonality, with most cases occurring during epidemiological weeks 35–46 each year (Figure 1).

### 3.2 Distribution of meteorological factors

During the study period, Tmax was 29.28 ± 3.88 °C (median, 30.11 °C), with values mainly ranging from 22.02 to 34.04 °C. DTRmean averaged 6.86 ± 1.23 °C (median, 7.00 °C). Hainan remained persistently humid, with a weekly mean relative humidity (RHmean) of 82.12 ± 5.09% (median, 82.37%). Mean sea level pressure (MSLP) was 758.50 ± 4.33 mmHg. Precipitation was highly skewed with episodic heavy rainfall: Pmean was 4.73 ± 6.99 mm/day (median, 1.79 mm/day), and the 95th percentile reached 16.74 mm/day. WSmean was 2.95 ± 0.58 m/s, and the weekly maximum gust (WGmax) averaged 14.03 ± 8.91 m/s (median, 17.00 m/s). Mean visibility (VISmean) was 19.02 ± 4.63 km (median, 18.95 km) (Table 1 and Supplementary Figure S3).

**Table 1:** Descriptive statistics for weekly meteorological variables in Hainan Province, 2018–2019 and 2023–2024.

Variable	Mean (SD)	Percentile				
		5	25	50	75	95
Tmax (°C)	29.28 (3.88)	22.02	26.80	30.11	32.44	34.04
RHmean (%)	82.12 (5.09)	74.47	79.33	82.37	85.84	89.09
MSLP (mmHg)	758.50 (4.33)	751.79	754.78	758.88	761.97	765.32
Pmean (mm/day)	4.73 (6.99)	0.01	0.45	1.79	6.26	16.74
WSmean (m/s)	2.95 (0.58)	2.00	2.57	2.91	3.24	4.10
WGmax (m/s)	14.03 (8.91)	0.00	11.00	17.00	20.00	24.00
VISmean (km)	19.02 (4.63)	11.96	14.95	18.95	23.01	26.39
DTRmean (°C)	6.86 (1.23)	4.99	6.01	7.00	7.70	8.78

Values are presented as mean (standard deviation) and selected percentiles (P5, P25, P50, P75, P95). Tmax: weekly mean daily maximum temperature; RHmean: weekly mean daily mean relative humidity; MSLP: weekly mean sea level pressure; Pmean: weekly mean daily precipitation; WSmean: weekly mean daily mean wind speed; WGmax: weekly maximum of daily maximum gust; VISmean: weekly mean of daily mean visibility; DTRmean: weekly mean diurnal temperature range.

### 3.3 Effects of meteorological factors on dengue risk

**Figure 2:**
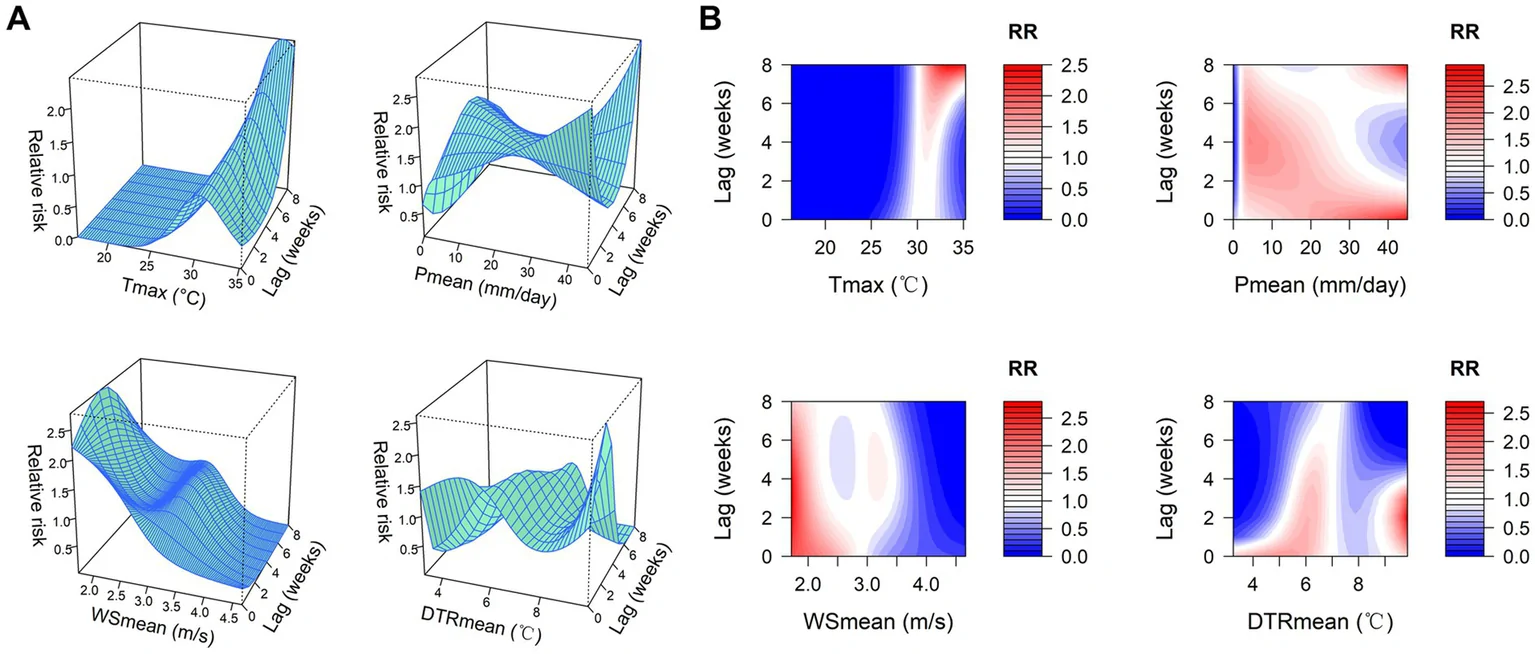
Lag-specific associations between meteorological variables and locally acquired dengue incidence in Hainan Province. **(A)** Exposure-lag-response surfaces. **(B)** Corresponding contour plots derived from DLNM. Relative risks were estimated using the median of each variable as the reference: 30.11 °C for Tmax, 1.79 mm/day for Pmean, 2.91 m/s for WSmean, and 7.00 °C for DTRmean.

All meteorological factors showed nonlinear and lagged associations with dengue risk (Figure 2). With the median as the reference, the *RR* of dengue was at or below 1 across all lags at lower temperatures, whereas at higher temperatures, risk increased mainly at longer lags, peaking at lag 8 when Tmax reached 33.73 °C (*RR* = 2.45, 95% CI: 1.70–3.63). Precipitation was protective at lower levels and harmful at higher levels, with risk peaking at lag 4 when Pmean was 4 mm/day (*RR* = 1.92, 95% CI: 1.59–2.31); at heavy rainfall (above ~28 mm/day) and lags of 5 to 6 weeks, the point estimates fell below 1, although this protective trend was not statistically significant. Wind speed showed an inverse association: low wind speeds increased risk, with the strongest effect at the lowest speeds (peaking at lag 3 at 1.72 m/s; *RR* = 2.77, 95% CI: 1.96–3.93), whereas high wind speeds were protective, with stronger protection at higher speeds. For DTRmean, a below-median diurnal temperature range increased risk, peaking at lag 0 at about 5 °C (*RR* = 1.74, 95% CI: 1.27–2.37), with the harmful effect persisting across the first few lags at moderate ranges (~6 °C); a larger range (above ~7 °C) was protective, with protection deepening at longer lags. Sensitivity analyses indicated that the main findings remained largely unchanged when the maximum lag period was varied (Supplementary Figure S4).

### 3.4 Effects of extreme meteorological conditions on dengue risk

**Figure 3:**
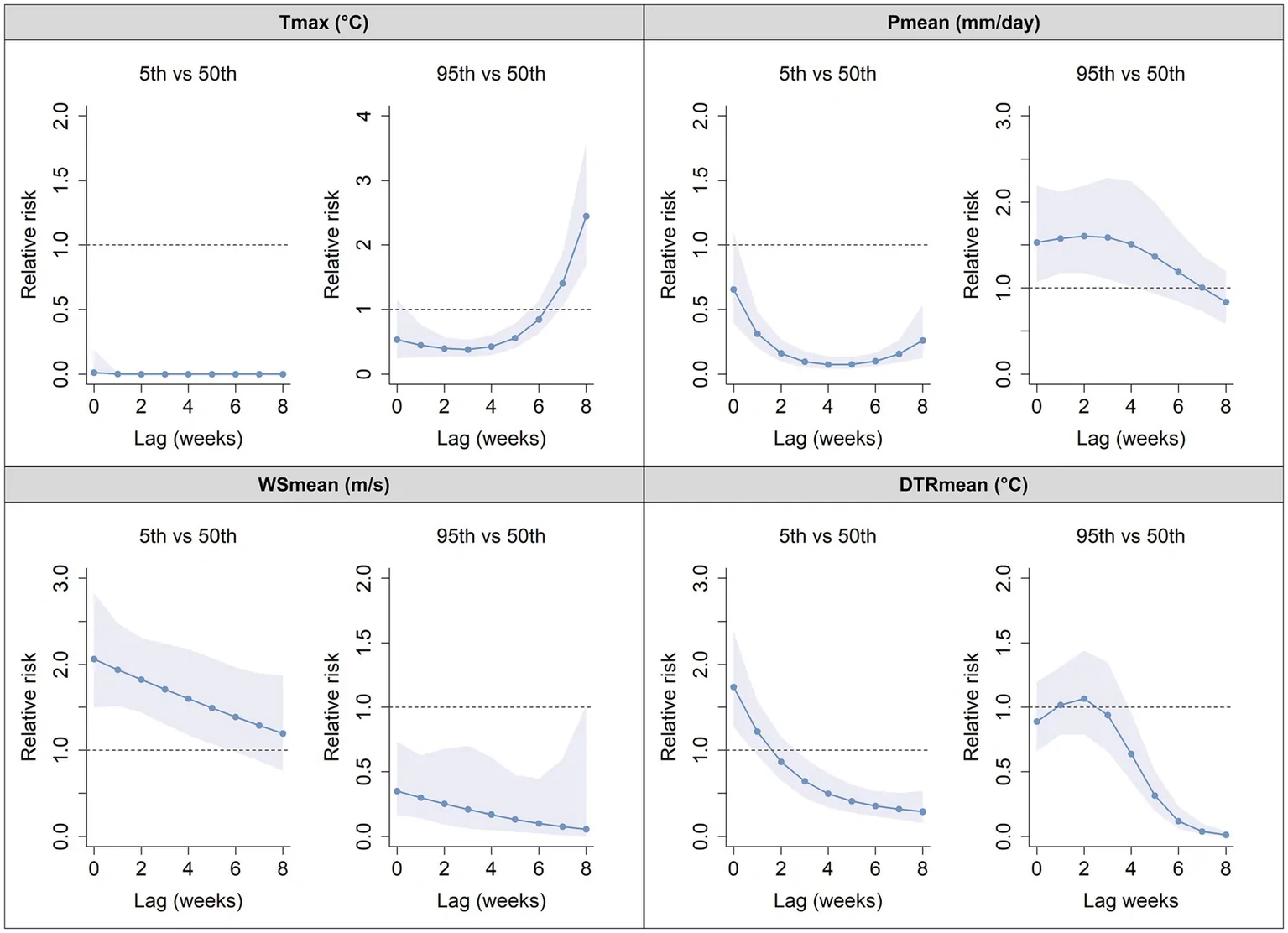
Lag-response relationships of extreme meteorological variables and locally acquired dengue incidence in Hainan Province. Extreme exposures were defined as the 5th (low) and 95th (high) percentiles of each meteorological variable, with median values used as references.

We contrasted the 5th and 95th percentiles of each meteorological variable (extremely low and high conditions) with the median (Figure 3).

Extremely low temperature and precipitation were strongly and consistently protective across all lags. Extremely low wind speed was associated with increased risk at lags 0 to 5, peaking at lag 0 (*RR* = 2.06, 95% CI: 1.50 to 2.83) and weakening thereafter. Extremely low DTRmean was harmful at lag 0 (*RR* = 1.74, 95% CI: 1.27 to 2.37) and became protective from lag 3 onward.

For extremely high temperature, the association was protective at lags 1 to 5 but harmful at the longest lags, peaking at lag 8 (*RR* = 2.45, 95% CI: 1.68–3.57). Extremely high precipitation increased risk at lags 0–4, with the strongest effect at lag 2 (*RR* = 1.60, 95% CI: 1.18–2.19). Extremely high wind speed was protective at lags 0–7, with protection deepening as lag increased. Extremely high DTRmean was protective at the longer lags (4–8), strengthening with lag and reaching its strongest effect at lag 8 (*RR* = 0.01, 95% CI: 0.003–0.04).

### 3.5 Cumulative lag effects of meteorological factors on dengue risk

**Figure 4:**
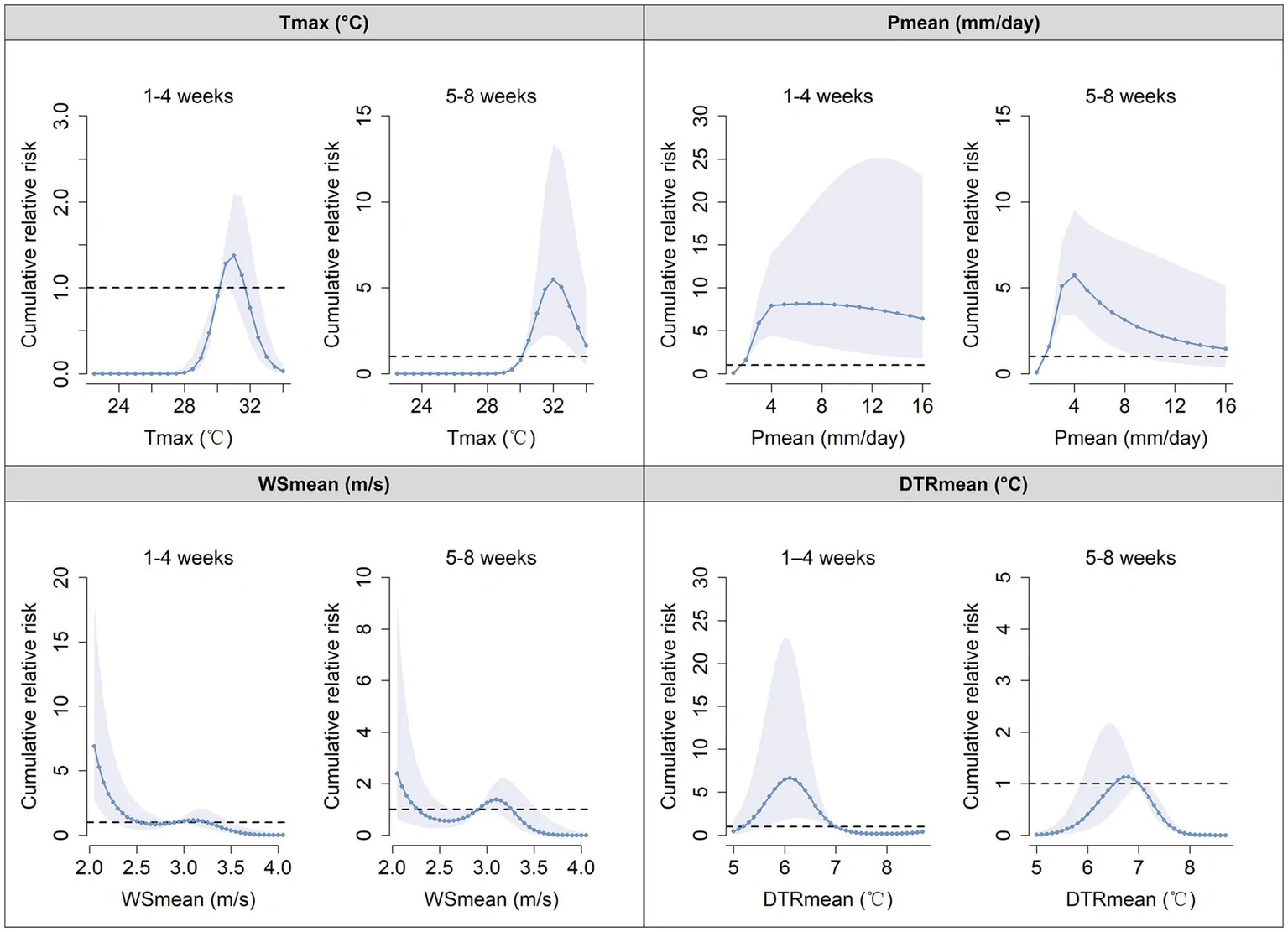
Cumulative exposure-response relationships between meteorological variables and locally acquired dengue incidence in Hainan Province. Associations are shown for cumulative lag windows of 1–4 and 5–8 weeks. The median exposure level of each meteorological variable was used as reference.

Over lags of 1 to 4 weeks, high temperature was modestly associated with increased cumulative risk, becoming significant and peaking at 30.5 °C (cumulative *RR* = 1.28, 95% CI: 1.04–1.58). Precipitation showed a strong cumulative effect that rose steeply and plateaued across about 4–10 mm/day (peak cumulative *RR* = 8.15 at 7 mm/day, 95% CI: 3.45–19.26). The cumulative risk of wind speed was highest at the lowest wind speeds (cumulative *RR* = 9.12 at 2.0 m/s, 95% CI: 3.46–24.00) and declined steadily, becoming protective above 3.55 m/s. DTRmean showed a unimodal cumulative association, peaking at 6.1 °C (cumulative *RR* = 6.64, 95% CI: 1.94–22.65) and turning protective above 7.1 °C (Figure 4).

Over lags of 5 to 8 weeks, the cumulative effect of high temperature increased markedly, peaking at about 32 °C with a much larger cumulative *RR* (cumulative *RR* = 5.48, 95% CI: 2.25–13.34). In contrast, the cumulative effects of the other variables were weaker than in the 1–4-week window: precipitation showed an inverted U-shaped association peaking at about 4 mm/day (cumulative *RR* = 5.73, 95% CI: 3.44–9.52); the harmful effect of low wind speed largely attenuated and lost significance, leaving only a marginal increase near 3 m/s; and DTRmean retained only a weak, non-significant peak around 6.8 °C while becoming significantly protective at higher values (Figure 4).

### 3.6 Dengue risk prediction

**Figure 5:**
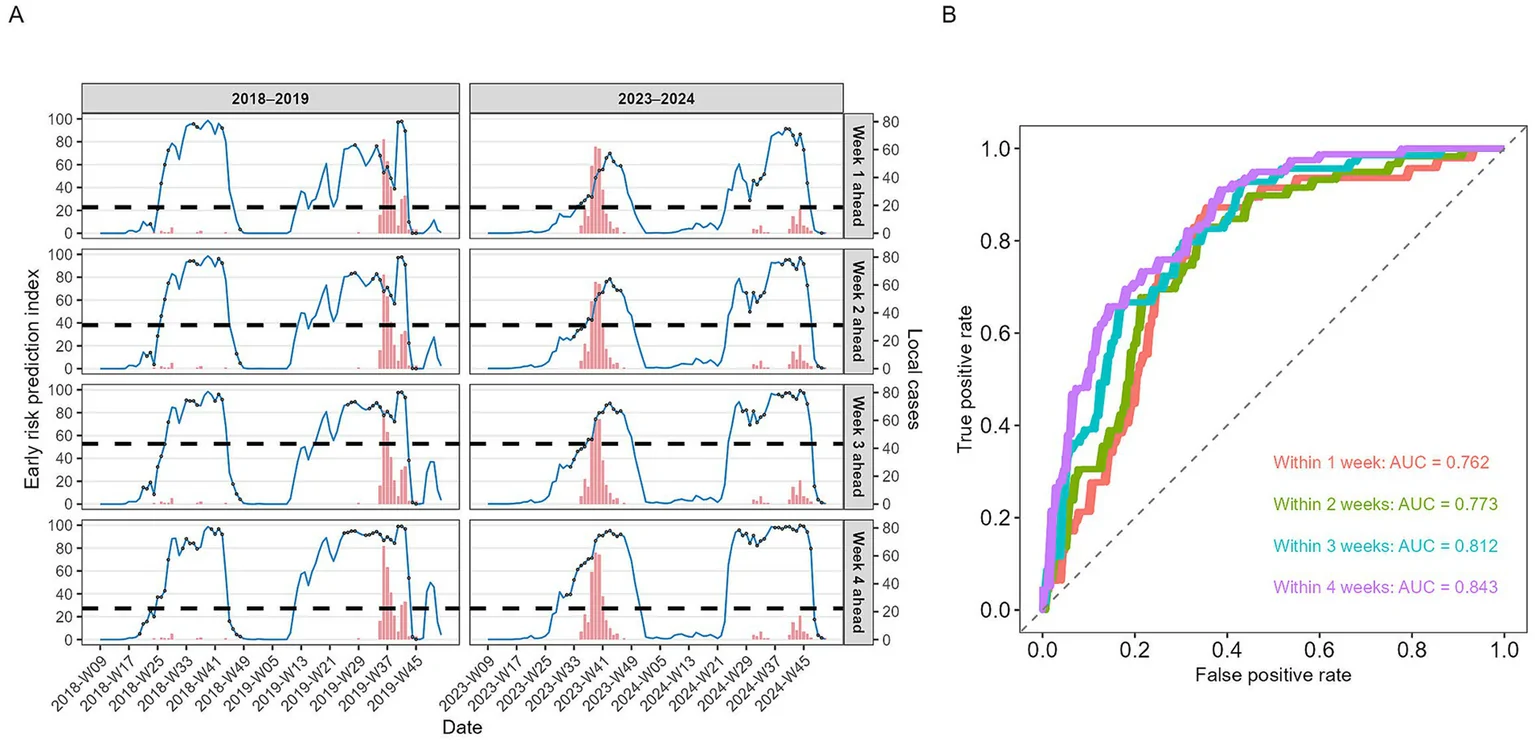
Temporal dynamics and performance of the ERPI for locally acquired dengue in Hainan Province. **(A)** Weekly ERPI values and observed locally acquired dengue cases. ERPI values range from 0 to 100 and represent the predicted risk score for the occurrence of at least one locally acquired case occurring anywhere in Hainan Province within the specified cumulative prediction window. **(B)** ROC curves for cumulative prediction windows of 1, 2, 3, and 4 weeks. ROC, receiver operating characteristic; AUC, area under the ROC curve.

We developed early risk prediction indices for dengue occurrence within the next 1, 2, 3, and 4 weeks to characterize short-term risk dynamics across forecasting horizons (Figure 5A). Across all four horizons, the risk index time series aligned well with the historical epidemic patterns of locally acquired cases, and increased in advance of multiple known dengue seasons. ROC analyses indicated good discriminative performance for predicting dengue occurrence within the subsequent month, with AUC values ranging from 0.762 to 0.843 for 1–4-week-ahead predictions (Figure 5B). Sensitivity remained relatively stable, ranging from 0.768 to 0.797, whereas specificity ranged from 0.644 to 0.708. PPV increased from 0.468 for the 1-week window to 0.624 for the 4-week window, while NPV decreased from 0.911 to 0.822 (Table 2). Furthermore, sensitivity analysis confirmed the robustness of our results. Altering the threshold for predicting dengue incidence did not significantly change our findings (Supplementary Figure S5 and Supplementary Table S1).

**Table 2:** Performance of the ERPI for locally acquired dengue at prediction horizons of 1–4 weeks in Hainan Province.

*k*	AUC	Sensitivity	Specificity	PPV	NPV
1	0.762	0.787	0.708	0.468	0.911
2	0.773	0.780	0.644	0.495	0.867
3	0.812	0.768	0.664	0.564	0.835
4	0.843	0.797	0.661	0.624	0.822

*k* denotes the cumulative prediction-window length in weeks. The outcome was the occurrence of at least one locally acquired dengue case anywhere in Hainan Province within the subsequent k weeks. AUC, area under the ROC curve; PPV, positive predictive value; NPV, negative predictive value.

## 4 Discussion

This study characterized the non-linear and lagged associations between meteorological factors and dengue transmission in Hainan, the largest tropical island in China, and explored their potential utility for dengue risk prediction. Meteorological factors showed differing temporal patterns: higher temperature was associated with increased dengue risk after a relatively long lag, with the strongest association observed at approximately 8 weeks; precipitation was mainly associated with risk over short-to-moderate lags; lower wind speeds tended to be associated with higher risk, whereas higher wind speeds were associated with lower risk; lower DTRmean showed the strongest association at lag 0. The index showed moderate-to-good discrimination for predicting dengue occurrence 1–4 weeks in advance, suggesting that meteorological information, together with imported case data, may provide useful signals for early risk assessment. These findings add to the evidence on the potential value of incorporating lag-specific meteorological signals into dengue risk prediction in Hainan.

High temperature was associated with an increased risk of dengue, with the risk peaking at a lag of 8 weeks, a pattern consistent with the temperature sensitivity of mosquito growth and development and of viral replication (6, 7). Higher temperatures can accelerate mosquito reproduction, development, and biting activity and shorten the extrinsic incubation period of the virus, thereby promoting dengue transmission over the subsequent weeks (35, 36). In Hainan, sustained local dengue virus circulation has not been established, and locally acquired outbreaks are generally initiated by imported viremic cases. Since dengue cannot spread directly from human to human, favorable meteorological conditions alone cannot spark local transmission; suitable temperature and rainfall only create ecological prerequisites for vector proliferation, which often precede notified indigenous cases by several weeks as mosquito populations expand and sustained transmission chains gradually become established (37). The relatively long lag observed in Hainan may reflect the cumulative time required for changes in vector abundance and competence, viral incubation within mosquitoes, human infection, and subsequent transmission amplification before increases in reported cases become apparent (36, 38, 39). In our study, a below-median maximum temperature was protective, which may be attributable to reduced vector activity and a prolonged viral incubation period (35). As temperature rose, the cumulative lagged risk increased and peaked at a maximum temperature of approximately 30.5 °C, beyond which the risk declined with further warming; this may reflect temperatures exceeding the optimal range for the vector, leading to reduced biological activity and survival (38). Xiang et al. (40) reported an optimal high-temperature range of 21.6–32.9 °C for dengue transmission in Guangzhou, which is broadly consistent with our findings.

Dengue risk peaked under moderate rainfall at a lag of about 4 weeks. The cumulative risk peaked at a mean precipitation of approximately 4 mm/day and declined as precipitation increased further. Moderate rainfall can create mosquito breeding sites and maintain humidity conducive to mosquito survival, intensifying dengue transmission over the subsequent weeks (41, 42). The approximately 4-week lag is biologically plausible because rainfall-induced increases in breeding-site availability must be followed by immature mosquito development, adult emergence, virus acquisition, the extrinsic incubation period, and subsequent human infection; a similar delay of approximately 27 days has been reported in southern China (8). At heavy rainfall, a declining trend in risk was observed (not statistically significant), plausibly because runoff flushes mosquito larvae and disrupts breeding sites while reducing outdoor activity and human-mosquito contact. Studies in Guangdong (43) and Singapore (44) reached consistent conclusions, confirming reduced dengue risk following heavy or extreme rainfall.

Wind speed showed a monotonic inverse association with dengue risk: risk was highest at low wind speeds and declined progressively as wind speed increased, becoming protective at high speeds, with the protective effect strengthening at longer lags. Calm or light-wind conditions favor *Aedes* flight, host-seeking, and biting, whereas high wind speeds physically impede mosquito flight and dispersal, reduce biting, and limit the spatial spread of dengue (45, 46). Previous time-series studies incorporating multi-week lags reached consistent conclusions, confirming a negative association between high wind speed and dengue risk (47).

Beyond temperature, temperature variability has been widely recognized as an important determinant of dengue transmission (10). In this study, a low mean diurnal temperature range increased dengue risk at short lags, whereas a range exceeding approximately 7 °C was protective. The immediate effect of a low range suggests that short-term temperature stability mainly influences adult mosquito activity and biting behavior, whereas large temperature fluctuations, which are often associated with frontal weather systems that temporarily disrupt the warm, humid conditions favorable to *Aedes* survival, can impose thermal stress on mosquitoes adapted to stable tropical climates, thereby reducing their survival and vectorial capacity (48–50).

Hainan lacks sustained autochthonous dengue virus circulation, and local transmission is generally initiated by imported cases (18, 20). Once a locally acquired case is detected, epidemiological investigations and vector control measures are rapidly implemented. Therefore, although the ERPI lacks spatial resolution and cannot identify transmission hotspots or directly guide resource allocation at the city or county level, it may still serve as a province-level early-screening signal, providing additional lead time to intensify case and vector surveillance, strengthen laboratory preparedness, and pre-position control supplies before local transmission is detected.

The ERPI achieved AUC values of 0.762–0.843 for predicting local dengue occurrence within the subsequent 1–4 weeks, with relatively stable sensitivity, supporting its potential value as a screening-oriented early warning tool. However, its PPV remained moderate, indicating that some high-risk alerts were not followed by a locally acquired case within the corresponding prediction window. The ERPI is therefore better suited to prompting risk verification and enhanced preparedness than to indicating that local transmission will necessarily occur, and a positive alert should not be used alone to trigger resource-intensive control measures. Nevertheless, this trade-off may be acceptable from a public health perspective because the consequences of missing an emerging outbreak may outweigh the costs of precautionary surveillance following a false-positive alert. As the prediction window increased from 1 to 4 weeks, NPV gradually declined, indicating that a negative alert was more informative for ruling out occurrence in the immediate future, but less reliable for excluding risk over longer periods. Overall, the ERPI should complement, rather than replace, routine epidemiological surveillance, imported-case monitoring, and vector surveillance. With the continued development of the Hainan Free Trade Port and increasing population mobility, the risk of local transmission initiated by imported cases may rise, further highlighting the need for prospective province-level risk warning (51).

Several limitations should be noted. First, the study covered only four epidemic years, with 2020–2022 excluded due to COVID-19 control measures. Such a short time series may destabilize nonlinear lag-effect estimation and limit our ability to detect longer-term climatic trends; additional validation is required once longer surveillance datasets become available. In addition, routine case surveillance tends to miss asymptomatic infections and patients who do not seek medical care, which may bias the estimated associations. Second, meteorological data and the ERPI were aggregated at the provincial level, which may induce ecological bias and obscure intra-provincial spatial heterogeneity. Additionally, the binary ERPI does not allow us to locate transmission hotspots or quantify outbreak severity, restricting its utility for city- and county-level vector control interventions. Third, this study did not incorporate several potential confounding factors, such as dengue interventions, entomological vector indices, population immunity, circulating viral serotypes, and socioeconomic and environmental factors (1, 22, 52). Accordingly, while the detected lag structures are consistent with biological mechanisms, the present analysis could not separately quantify the independent effects of vector dynamics, viral incubation, transmission amplification and control measures. Finally, the ERPI was neither benchmarked against conventional statistical or machine-learning models nor externally validated. Its predictive accuracy and practical value thus require further rigorous evaluation.

## 5 Conclusion

This study characterized the non-linear, lagged, and cumulative associations of meteorological factors with local dengue transmission in Hainan Province. Higher temperature drove medium- to long-term dengue risk, while a low-to-moderate diurnal temperature range increased short-term risk. Precipitation was associated with risk across both short and medium-to-long lags, while cumulative wind-speed associations were strongest over shorter lags and attenuated thereafter. The DLNM-based early risk prediction index, integrating meteorological monitoring with epidemiological surveillance and may support early dengue risk assessment and prevention efforts in tropical settings such as Hainan. External validation is nevertheless required before operational implementation of the index.

## Data Availability

The data analyzed in this study is subject to the following licenses/restrictions: dengue surveillance data for Hainan Province were obtained from the China CDC National Notifiable Disease Reporting System. These data are not publicly available due to data-use restrictions but can be requested from the Chinese Center for Disease Control and Prevention upon reasonable application. Requests to access these datasets should be directed to https://www.phsciencedata.cn/Share/index.jsp.

## Footnotes

1
https://rp5.ru/
