# Supplementary material for: Meteorological drivers and short-term prediction of dengue fever in a tropical region of China: evidence from Hainan Province
Source: Front Public Health. 2026 Aug 28;14:1919479. doi: 10.3389/fpubh.2026.1919479 (PMC13561798; doi:10.3389/fpubh.2026.1919479)
Supplement: Supplementary file 1 [file Supplementary_file_1.DOCX]

Supplementary Material

**Supplementary Figures**

**Figure S1.** Lagged Spearman correlations between meteorological variables and locally acquired dengue cases in Hainan Province

**Figure S2.** Spearman rank correlations among meteorological variables in Hainan Province

**Figure S3.** Weekly time series of meteorological variables in Hainan Province, 2018–2019 and 2023–2024

**Figure S4.** Sensitivity analysis of lag-specific associations between meteorological variables and locally acquired dengue incidence

**Figure S5.** Sensitivity analysis of the temporal dynamics and predictive discrimination of the ERPI in Hainan Province

**Supplementary Tables**

**Table S1.** Sensitivity analysis of performance of the ERPI in Hainan Province

**Figure S1. Lagged Spearman correlations between meteorological variables and locally acquired dengue cases in Hainan Province. Spearman** correlation coefficients were calculated at different weekly lags. Tmean: weekly mean of daily mean temperature; Tmin: weekly mean of daily minimum temperature; Tmax: weekly mean of daily maximum temperature; Pmean:weekly mean daily precipitation; MSLP: weekly mean sea level pressure; VISmean: weekly mean of daily mean visibility; RHmean: weekly mean of daily mean relative humidity; WSmean: weekly mean of daily mean wind speed; WGmax: weekly maximum of daily maximum gust; DTRmean: weekly mean diurnal temperature range; LC: locally acquired dengue cases; IC: import dengue cases.

**Figure S2. Spearman rank correlations among meteorological variables in Hainan Province.** Spearman rank correlation coefficients are shown in each cell. Cell colours indicate the direction and magnitude of correlations. *P < 0.05; ** P < 0.01; *** P < 0.001; NS: non-significant correlations (P > 0.05).

**Figure S3. Weekly time series of meteorological variables in Hainan Province, 2018–2019 and 2023–2024.** Tmean: weekly mean of daily mean temperature; Tmin: weekly mean of daily minimum temperature; Tmax: weekly mean of daily maximum temperature; Pmean:weekly mean daily precipitation; MSLP: weekly mean sea level pressure; VISmean: weekly mean of daily mean visibility; RHmean: weekly mean of daily mean relative humidity; WSmean: weekly mean of daily mean wind speed; WGmax: weekly maximum of daily maximum gust; DTRmean: weekly mean diurnal temperature range.

**Figure S4.** **Sensitivity analysis of lag-specific associations between meteorological variables and locally acquired dengue incidence.** Associations were estimated using (A) the primary model specification, with a maximum lag of 8 weeks; (B) an alternative maximum lag of 7 weeks; and (C) an alternative maximum lag of 9 weeks.

**Figure S5. Sensitivity analysis of the temporal dynamics and predictive discrimination of the ERPI in Hainan Province.** The outcome was the occurrence of at least three locally acquired dengue cases anywhere in Hainan Province. (A) Weekly ERPI values and observed locally acquired dengue cases. ERPI values range from 0 to 100 and represent the predicted risk score for the occurrence of at least three locally acquired cases occurring anywhere in Hainan Province within the specified cumulative prediction window. (B) ROC curves for cumulative prediction windows of 1, 2, 3, and 4 weeks. ROC: receiver operating characteristic; AUC: area under the ROC curve.

**Table S1. Sensitivity analysis of performance of the ERPI in Hainan Province**

| ***k*** | **AUC** | **Sensitivity** | **Specificity** | **PPV** | **NPV** |
| --- | --- | --- | --- | --- | --- |
| 1 | 0.790 | 0.688 | 0.767 | 0.373 | 0.924 |
| 2 | 0.810 | 0.730 | 0.747 | 0.409 | 0.920 |
| 3 | 0.821 | 0.833 | 0.732 | 0.467 | 0.940 |
| 4 | 0.836 | 0.787 | 0.764 | 0.521 | 0.917 |

*k* denotes the cumulative prediction-window length in weeks. The outcome was the occurrence of at least three locally acquired dengue cases anywhere in Hainan Province within the subsequent k weeks. AUC: area under the ROC curve; PPV: positive predictive value; NPV: negative predictive value.
